# O-15 - DEVELOPING A NATIONAL SURVEILLANCE SYSTEM FOR RECORDING ENVIRONMENTAL INCIDENTS OF PUBLIC HEALTH CONCERN IN IRELAND

**DOI:** 10.1093/pubmed/fdag046.014

**Published:** 2026-07-09

**Authors:** Ms Mairead Madigan, Mrs Patricia Garvey, Ms Angeline McIntyre, Ms Aoife Colgan, Ms Ina Kelly, Environmental Incidents sub-group of the National All-Hazards Group (EISNAHG) Yvonne Williams, Emer O Connell, Breda Cosgrove, Orla Bruton, Aoife Colgan, Patricia Garvey, Ina Kelly, Mary T O'Mahony, Mette Jensen, Edel Evans, Patricia Carney, Mary O' Meara, Ruth McDermott

**Affiliations:** Health Protection Surveillance Centre; Health Protection Surveillance Centre; Health Protection Surveillance Centre; Health Protection Surveillance Centre; National Health Protection Office; Environmental Incidents sub-group of the National All-Hazards Group (EISNAHG)

## Abstract

**Background:**

Environmental hazards are established health threats. Environmental Incidents (EI) of Public Health concern are reported to Regional Departments of Public Health from a variety of sources in Ireland. A 2023 review of regional data showed that inclusion criteria for incidents differed between departments and were not standardised. Only regional data was collected.

**Objectives:**

Our aim was to develop and pilot a national EI surveillance system to support the collection, monitoring, and reporting of EI in Ireland. It will inform recommendations for Outbreaks, Case and Incident Management System (OCIMS), the new system which will manage the surveillance of infectious diseases and public health threats in Ireland.

**Methods:**

Between September 2024 and January 2025, consultations with regional public health colleagues informed incident types and key variables. Further meetings after one quarter of data collection gathered feedback on EI recording. Pilot data collected between the 1st April to 30th September 2025 were analysed in RStudio.

**Results:**

The national EI surveillance system was created using an excel database with nine EI types, a core suite of variables and issued to Regional Departments of Public Health for data entry. In a pilot between 1st April to 30th September, 234 incidents were recorded. Drinking water (DW) accounted for 69% (n=162) of EI. 60% of DW incidents were linked to public water supplies. Bathing water (BW) accounted for 25% of EI, 79% of which were associated with sea/salt water. Advice was required for the public for 93% of EI, including 43 boil water notices, and 40 bathing prohibition notices.

**Conclusions:**

This pilot constitutes Ireland’s first national EI surveillance system of public health responses to EI, allowing description of incident patterns, assessment of recommendations, and identification of gaps. It also led to new variables for sub-categorising DW incidents and public health advice, and informed recommendations for OCIMS.

